# Spatial aggregation of fruits explains food selection in a neotropical primate (*Alouatta pigra*)

**DOI:** 10.1038/s41598-019-55932-y

**Published:** 2019-12-19

**Authors:** John F. Aristizabal, Simoneta Negrete-Yankelevich, Rogelio Macías-Ordóñez, Colin A. Chapman, Juan C. Serio-Silva

**Affiliations:** 1 Red de Biología y Conservación de Vertebrados, Instituto de Ecología A.C., Carretera antigua a Coatepec No. 351, El Haya, CP 91070 Xalapa, Veracruz, México; 2 Red de Ecología Funcional, Instituto de Ecología, A.C., Carretera antigua a Coatepec No. 351, El Haya, Xalapa, Veracruz, 91070 México; 3 Red de Biología Evolutiva, Instituto de Ecología, A.C., Carretera antigua a Coatepec No. 351, El Haya, CP 91070 Xalapa, Veracruz, México; 4 Department of Anthropology, Center for the Advanced Study of Human Paleobiology, The George Washington University, Washington, DC 20037 USA; 5 School of Life Sciences, University of KwaZulu-Natal, Scottsville, Pietermaritzburg, South Africa; 6 Shaanxi Key Laboratory for Animal Conservation, Northwest University, Xi’an, China

**Keywords:** Behavioural ecology, Ecological modelling

## Abstract

The availability and spatial distribution of food resources affect animal behavior and survival. Black howler monkeys (*Alouatta pigra*) have a foraging strategy to balance their nutrient intake that involves mixing their consumption of leaves and fruits. The spatial aggregation of food items should impact this strategy, but how it does so is largely unknown. We quantified how leaf and fruit intake combined (here termed *food set selection*) was spatially aggregated in patches and how food aggregation varied across seasons. Using variograms we estimated patch diameter and with Generalized Least Square models determined the effect of food spatial aggregation on food selection. Only fruits were structured in patches in the season of highest availability (dry-season). The patches of food set selection had a diameter between 6.9 and 14 m and were explained by those of mature fruit availability which were between 18 and 19 m in diameter. Our results suggest that the spatial pattern of food selection is influenced by patches of large fruit-bearing trees, not by particular species. Fruit also occur along spatial gradients, but these do not explain food selection, suggesting that howlers maximize food intake in response to local aggregation of fruit that are limiting during certain seasons. We demonstrate how the independent spatial modelling of resources and behavior enables the definition of patches and testing their spatial relationship.

## Introduction

Food resources generally have an aggregated spatial distribution^1^. Therefore, the spatial distribution of diversity, abundance, and behavior of animals that consume such resources are neither uniform nor random^1^. Typically, spatial modeling of animal behavior and feeding patterns have been carried out at a landscape scale^2,3^. At this scale, the effects of spatial variation (e.g., fragment size, forest cover) influence habitat selection and animal behavior^4,5^. However, smaller scales variation can also have an effect. For example, Razgour *et al*.^6^ documented that habitat selection by a bat (*Plecotus austriacus*) has different drivers on broad scales (limited by inadequate climatic conditions) than on small scales (limited by the availability of preferred foraging areas). Jedlikowski *et al*.^7^ studied habitat selection of two rallid birds at three spatial scales, landscape (50–300 m), territory (14 m), and nest (3 m), and found that bird occurrence was most associated to the territory scale as they make foraging decisions at that scale. In socioecological studies of agonism in primates, it has been suggested that the feeding area that an individual can defend is dependent on body size at small scales^8^. Small species typically defend small foraging areas^8^.

Primates are ideal to examine the effect of spatial scale on behavior and distribution as observations can be carried out on each individual in a group and can include the behavioral variability among groups and populations. The time spent resting and eating leaves by spider monkeys (*Ateles geoffroyi*) were mostly correlated with the forest cover at the 126-ha scale; these correlations were lower when broader scales were considered^3^. The 126-ha scale is similar to their home ranges, suggesting that results will often depend on the chosen scale for ecological studies^9,10^. Understanding foraging strategies at small spatial scales is important to understand the consequences of habitat loss, as fragmentation forces animals to typically only make decisions at this scale^11^.

Spatial statistics are useful for evaluating the distribution of ecological phenomena and can test hypotheses on how habitat quality influences animal behavior^12–15^. The choice of the scale at which data are recorded defines which *spatial structures* and *patterns* (i.e., indicated by spatial autocorrelation) can be detected and the ability to estimate their degree of *aggregation* (formally defined as the amount of variance explained by the spatial pattern)^16^. The study’s spatial *scale*, encompasses three elements: the *grain*, which is the size of an observation, the sampling *interval*, the minimum distance between the grains, and the *extension*, which is the total area of the study^16,17^. Ecological phenomena and their spatial structures can be detected in two forms. *Patche*s are areas where the values of the variables are more similar or *autocorrelated* than randomly expected. *Gradients* or trends are those phenomena at broader scales, detectable as gradual changes of a variable throughout the area. These structures contrast with *random* spatial fluctuations^16,17^.

The spatial patterns and phenology of trees in tropical forests determine the distribution and periodicity of food items for frugivores and folivores^18,19^. These items have a generally uniform and sparse production most of the year^19^. Peaks in the production of fruits and young leaves occur in dense and spatially delimited areas for short periods^9,20,21^. Evidence suggests the measuring resource availability and feeding behavior at biologically relevant scales must be made from the perspective of individuals^8^. A reduction in the availability of food with needed nutrients can reduce birth rates, increase mortality rates of young, and intensify diseases^22^. Availability of young leaves and fruits are particularly important for primate species due to their low fiber and toxin concentration and their high nutrient and water contents^21,23–25^. Many studies of arboreal primates have related food variables (e.g., food density and temporal availability) to the animals location^3,18,26,27^. A common method to quantify seasonal food availability is to monitor the phenophases of a subsample of each tree species in an area and then weight the proportion of individuals providing each food item by the density of that species^20,28,29^.

Traditionally, individual trees have been used as the study unit to measure food choice in arboreal primates^20,30,31^. However, to answer many questions the forest canopy should be conceived as a continuum in space where the aggregation of resources does not necessarily reflect discrete individual tree identity^8,32^. Food selection also depends on other aspects such as nutrient balancing^8,33^, competition^34^, and the distribution of several discrete and depletable food patches (i.e., groves of food trees)^29^. Food patches for arboreal primates are commonly defined in two ways: (1) the size of a single tree, indexed by diameter at breast height (DBH)^20,35^, or (2) the distance between food patches or a group of food trees of the same species with adjoining canopies^31^. For instance, tamarin monkeys (*Saguinus mystax*) use large patches (food aggregation areas made up by different trees) or small patches (a single tree) depending on competition for resources with other primate species^34^. Black howler monkeys (*Alouatta pigra*) present a resource mixing strategy of leaves and fruits and they frequently change patches defined as a single feeding tree from one particular food type^35^. Nevertheless, patch is almost always defined without any statistical estimation of the patch’s diameter or confirmation of the aggregation significance. Without a statistical consideration it is not known if the limits of the foraging patches coincide with the aggregation of food and the animal’s foraging decisions^8,32,36^. Thus, it is not clear how the spatial distribution of a particular item, or the food items consumed together to obtained a balanced diet (e.g., fruit and leaves combined), influences its selection. We use the term *food set selection* for this combined intake of food items.

Here we evaluated the spatial structure of leaf and fruit availability and the selection of food items in black howler monkeys (hereafter howlers). This was done within two fragments (<2.1 ha) of tropical forest in Mexico. We modeled *gradients* and *patches* of fruit and leaf availability in different seasons to test three hypotheses. 1) The spatial structuring in patches associated to fruits and leaves would have a greater degree of aggregation in seasons of greater availability. 2) Given the resource mixing strategy of howlers, the food set selection would be spatially aggregated on a patch diameter and a gradient direction similar to those associated to the availability of the preferred and limiting resources, mature fruits or young leaves in seasons of greater availability. 3) The food set selection, considered in terms of patches and gradients, would be explained by the aggregation of mature fruits and young leaves in their respective season of greater availability.

## Materials and Methods

### Study site

Black howler monkeys were studied in forest remnants around the “La Estación de Investigación Primatologica y de Vida Silvestre” in the state of Tabasco, Southeast Mexico. The landscape consists of forest fragments within pasture and cattle farms and includes tropical dry forest, palm forest, riverine forest, and secondary forest. The average monthly temperature ranges between 21.7 °C and 33.5 °C. There are three distinct seasons: a rainy season from May to October with an average rainfall of 1,780 mm, a “nortes” season characterized by strong winds from the North from November to January and an average rainfall of 1,370 mm, and a dry season from February to April with an average rainfall of 570 mm^37^.

**Figure 1:**
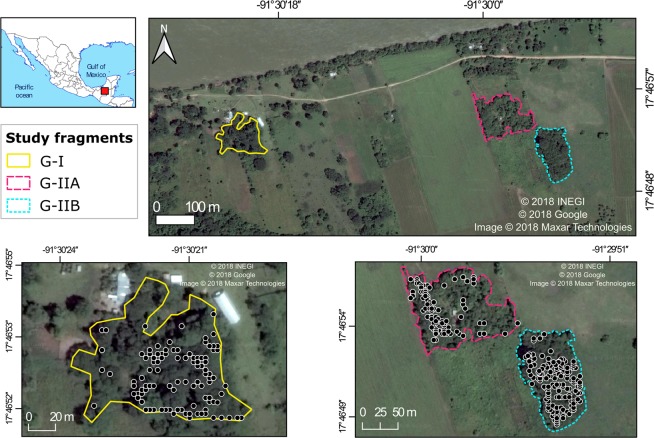
Location of the study fragments and trees inside each fragment (black circles). Remote sensing image of the study area generated using Google Earth Pro 7.1.8.3036 (https://earth.google.com/web/) and QGIS software (http://qgis.osgeo.org)^43^.

We studied groups in two fragments separated by 500 m; Group-I (G-I) lived in a fragment of 0.8 ha and Group-II (G-II) occupied a fragment of 2.1 ha (Fig. 1). We divided the G-II forest fragment in a northern site (1.0 ha, G-IIA) and a southern site (1.1 ha, G-IIB) since its “8” shape disrupts the spatial continuity required for spatial analyses (Fig. 1). We carried out a census of all trees (diameter at breast height-DBH ≥10 cm) in both fragments and located and labeled all trees (G-I: 142 trees, G-IIA: 77, G-IIB: 156). We identified all trees to species level using reference material from the INECOL herbarium collection (XAL). Each month we visually assessed food availability of all trees and scored the abundance of vegetative (young and mature leaves) and reproductive parts (mature and immature fruits, and flowers) on a zero to five scale. With this we assigned phenological categories (PC) according to the percentage of the crown containing food item: 0 = 0%, 1 = 1–25%, 2 = 26–50%, 3 = 51–75%, 4 = 76–100%. We calculated the importance value index (IVI) as a measure of availability for each tree species. This is calculated by adding the density, relative abundance, and dominance of each tree species (*sp*) for each fragment (modified from Agostini *et al*.^38^; Salomao *et al*.^39^):

1 $$IV{I}_{sp}=\frac{{N}_{sp}}{{S}_{f}}+\frac{{N}_{sp}}{{N}_{t}}+{\sum }_{i=1}^{n}\frac{B{A}_{i}}{B{A}_{tf}}$$

where *N*_*sp*_ is the number of trees of *sp* species, and *S*_*f*_ is the area of fragment *f*; *N*_*t*_ is the total number of trees in the fragment. *BA* is the basal area of each *i* species (ranging from 1 to *n* marked trees of the same species), or of *tf*, all trees in the fragment added.

### Activity and feeding of black howler monkeys

We followed two groups of howler monkeys from June 2016 to May 2017 completing 106 days of ≈12 hr of daily observations. Group-I (G-I: 426.9 hours) had 1 adult male, 2 adult females, 2 infants. Group-II (G-II: 433.1 hours) had 1 adult male, 2 adult females, 1 infant. We followed six adult focal animals, each during two days per individual per month for each group. We recorded the time invested: *feeding*, *resting*, *traveling*, and *other* (i.e., playing, vocalization, and agonistic encounters). We recorded the time devoted to the consumption of each of the five food categories that constitute >90% of howlers’ diet^40^: *mature leaves*, *young leaves*, *mature fruits*, *immature fruits*, and *flowers*. We estimated the dry weight intake in grams of the items^41^. For this, we calculated a feeding rate for each item by counting the number of food units eaten per minute. To calculate dry weight intake, we collected food items from the tree crowns using 15-m poles and weighed 100 units of each item to estimate the fresh weight mass consumed within a maximum of five days from the first observed consumption of a new food. Subsequently, we collected between 150 and 250 g of fresh material per food item, and dried the samples in a cool, dark room at room temperature (ranges between 21.7 °C and 33.5 °C). To avoid the fermentation and molding of fruits, we used a food dehydrator (NESCO-FD-2000) at 55 °C until the sample reached a constant weight.

### Food availability and selection

We calculated two indices of food availability.

*Intraspecific index of Food Availability* (IFA) measures the availability of food items weighted by the size of the tree:

2 $$IF{A}_{Ia}={\overline{PC}}_{I}\times DB{H}_{a}$$

where the IFA value of an *I* food item of the tree *a* is equal to the average of the ${\overline{PC}}_{I}$ phenological category of the item in a given season for the tree *a*, multiplied by its DBH_*a*_.

*Interspecific index of Food Availability* (IDA) measures the availability of food items per tree given the importance value of the species (equivalent to FAI index used in Agostini *et al*.^38^ and Chaves and Bicca-Marques^28^):

3 $$ID{A}_{Ia}={\overline{PC}}_{I}\times IV{I}_{sp}$$

where the IDA value of an *I* food item is equal to the average of the ${\overline{PC}}_{I}$ phenological category of the item in a given season for the tree *a*, multiplied by the importance value index (IVI) of the species (*sp*) to which that tree belongs^39^. Note that the estimation of dominant species through the IVI index uses the absolute tree density. In our study this is constant because all tree species in a plot share the same area. However, we have left this component to make IDA applicable to situations where sites with different basal areas and tree species composition are being compared in terms of animal choices. While IFA gives greater weight to the contribution of large trees in the availability estimate, IDA magnifies the contribution of trees if they belong to one of the dominant species in the fragment. Using both indexes allowed us to consider the effect on food item availability by individual tree variation from the effect due to the importance of some species in the fragment.

Finally, we evaluated the food selection of each food item type separately and by food set (leaf and fruit intake combined, i.e., the sum of all items consumed) for of each tree by adding feeding bouts:

4 $$S{F}_{Ia}={\sum }_{i=1}^{n}{{\rm{FB}}}_{a}$$

where SF (selected food) intake of the item *I* in a given season is the sum of the dry weight intake in all feeding bouts FB (from *i*=1 to *n* bouts) of this item from the tree *a*.

### Data analyses

#### Spatial structuring of food availability and selection

The scale of the study is defined by *grain*, *extension*, and *range*, but only *extension* differed between the fragments^16,42^. Extension was 0.8 ha for G-I site, 1.0 ha for G-IIA site and 1.1 ha for G-IIB site (calculated using geometry option of QGIS^43^). For all three sites, the *grain* was 3 m corresponding to the average diameter of tree crowns. The *interval* was 0.5 m, the minimum distance between trees. We evaluated the spatial structuring of food availability modeling the following variable combinations (96 models): 2 availability indexes (IFA, IDA) x 4 food items (YL, ML, MF, IF) x 4 seasons (Rainy, Nortes, Dry, Annual) x 3 Sites (G-I, G-IIA, G-IIB). The spatial structuring of selected food (SF) was evaluated using the following combinations (60 models): 5 food items (YL, ML, MF, IF, Food-Set) x 4 seasons (Rainy, Nortes Dry, Annual) x 3 Sites (G-I, G-IIA, G-IIB). The spatial modeling method (and its R code) used in this study is described in detail in Negrete-Yankelevich and Fox (2015)^16^; however, we briefly summarize it here. Spatial modelling was done in two steps, modeling of *gradients* followed by *patches*^16^. We log or root-transformed the variables to comply with the assumption of normality of the residuals of linear modeling (see Table 1 for variable-specific transformations).

**Table 1:** Parameters of gradient models and variograms describing the spatial structures of the studied variables of black howler monkey groups.

Group/Variable	Gradient model				Variogram model				
Transformation [Item_Season]	α (int)	β (y)	γ (x)	ΔAIC	Model	Range (m)	Nugget	Sill	R^2^
**G-I SITE**									
**IFA-intraspecific index of food availability**									
Log_10_[Mature leaves_Nortes]	4.28	ni	0.0098	5	Spherical	11.9	0.251	0.34	0.3
Log_10_[Young leaves_Rainy]	3.9	ni	0.004	5	Spherical	15.2	0.255	0.35	0.5
Log_10_[Immature fruits_Year]	ni	ni	ni	ni	Spherical	54.7	0.152	0.25	0.7
Log_10_[Immature fruits_Dry]	ni	ni	ni	ni	Exponential	57.6	0.008	0.17	0.5
Log_10_[Mature fruits_Year]	ni	ni	ni	ni	Spherical	20.8	0.054	0.257	0.5
Log_10_[Mature fruits_Dry + 0.001]	1.17	0.007	−0.006	8	Spherical	18.0	0.051	0.148	0.3
**IDA-interspecific index of food availability**									
^3^√[Mature leaves-Rainy]	ni	ni	ni	ni	Spherical	17.5	0.52	0.83	0.4
√[Immature fruits_Dry + 0.5]	11.13	−0.008	ni	5	Spherical	6.7	0.143	0.436	0.3
^4^√[Mature fruits-Year]	ni	ni	ni	ni	Spherical	6.9	0.05	0.157	0.4
Log_10_[Mature fruits-Dry + 1.5]	ni	ni	ni	ni	Spherical	26.1	0.086	0.134	0.3
**SF- Selected food (gr)**									
Log_10_[Food set_Year]	2.121	0.001 (Y^2^)	ni	6	—	—	—	—	—
^4^√[Food set_Dry]	ni	ni	ni	ni	Spherical	14.0	0.044	1.31	0.3
Log_10_[Mature fruits_Year]	1.91	0.0018 (Y^2^)	ni	5	—	—	—	—	—
Log_10_[Mature leaves_Rainy + 0.1]	ni	ni	ni	ni	Spherical	10.3	0.49	3.02	0.3
^3^√[Mature leaves_Dry]	0.48	−0.031	ni	5	Exponential	74.1	0.59	1.522	0.4
**G-II SITE**									
**A site_IFA-intraspecific index of food availability**									
[Mature fruits_Year]	ni	ni	ni	ni	Spherical	35	39.2	61.4	0.6
√[Mature fruits_Dry + 10]	16.04	0.022	ni	5	Spherical	19.5	1.31	2.31	0.5
√[Immature fruits_Year + 1]	ni	ni	ni	ni	Spherical	20.3	1.6	2.7	0.4
**B site_IFA-intraspecific index of food availability**									
√[Young leaves_Nortes]	1.13	0.0068 (Y^2^)	ni	5	—	—	—	—	—
√[Immature fruits_Year]	5.96	−0.019	ni	ni	—	—	—	—	—
Log_10_[Mature fruits_Year + 1]	ni	ni	ni	ni	Spherical	40.3	0.195	0.51	0.7
Log_10_[Mature fruits_Dry + 1]	ni	ni	ni	ni	Spherical	40.9	0.19	0.58	0.7
**A site_IDA-interspecific index of food availability**									
^3^√[Mature fruits_Dry]	3.29	0.008	ni	5	Spherical	18.9	0.185	0.338	0.5
^3^√[Immature fruits_Year]	ni	ni	ni	ni	Spherical	17.3	0.28	0.43	0.3
**B site_IDA-interspecific index of food availability**									
√[Mature fruits_Year + 1]	3.55	−0.011	ni	7	Spherical	41.8	0.875	1.56	0.5
√[Mature fruits_Dry]	ni	ni	ni	ni	Spherical	37.3	2.16	3.73	0.5
**A site_SF_Selected food (gr)**									
^4^√[Food set_Nortes]	1.69	0.014	ns	6	—	—	—	—	—
^4^√[Food set_Dry]	ni	ni	ni	ni	Spherical	6.9	0.16	2.32	0.3

IFA-intraspecific index of food availability = $\overline{PC}\times {\rm{DBH}}$, where $\overline{PC}$ is the average of the phenological scores. IDA: Interspecific index of food availability = $\overline{PC}\times {\rm{IVI}}$, where IVI is the importance value index of trees. SF = Selected food (grams of dry weight). Food set = leaf and fruit intake (i.e. the sum of all items consumed). α, β and γ denote the estimate parameters for intercept, the north-south and east-west coordinates, respectively. ΔAIC = AIC_null_ - the AIC_selected model_ (AIC_null_ is the AIC of the response variable explained by its mean). ni = not included in the model after model selection. A site and B site correspond to the G-II´s fragment division (North and south, respectively). See variograms in Supplementary Fig. S3.

To detect possible *gradients* (i.e., monotonic changes in a given direction), we fitted linear models using north-south (*y*) and east-west (*x*) coordinates of each tree as explanatory variables. Note that these models are descriptive (i.e., not hypothesis tests), therefore the spatial independence in the data is not required^16^. To consider different forms of gradients (monotonic changes to a given direction or combinations of directions) we started the modeling of variables including the combination of different polynomial forms of their position in space as explanatory variables (*x* + *y* + *x*^2^ + *x* × *y* + *y*^2^). We simplified the models by successively eliminating the components of *x* and *y* from the polynomial equation. Variables were removed in increasing order of the estimated slope. The change Akaike information criterion (ΔAIC = AIC_null_ - the AIC_model_) was calculated for the model with and without the variable being examined. AIC_null_ was the AIC of the model where the response variable is solely explained by its mean. In each step, the model with the highest ΔAIC was kept before proceeding to the elimination of the next variable. We considered the existence of a gradient if at the end of the simplification the model included an *x* or *y* explanatory variable and had a ΔAIC greater than five units^44^.

To model the *patch* structure of the variables, we used variography. This is a method used to estimate the average size of patches present in the residuals of the linear models in the first step, once the variance explained by the gradient has been eliminated. Experimental variograms were constructed, which are graphs of semi-variance (an inverse measure of the autocorrelation) as a function of separation distance between pairs of observations^16,17^. We calculated:

5 $$\gamma (h)=\frac{1}{2W(h)}{\sum }_{i=1}^{W(h)}{[{y}_{i}-{y}_{i+h}]}^{2}$$

where *γ (h)* is the semi-variance of all pairs of observations located at *h* distance (lag*), W(h)* is the total number of observation pairs separated by *h*, and *y*_*i*_ is the value of the sample at each location, tree position in this case. We followed the recommendation to construct the variograms using pairs of observations that were <2/3 of the observed maximum distance (60 m) between observed points^16^. We did this to avoid including distances represented by a small number of pairs which could bias the estimates of the theoretical models that were subsequently fitted^16^. Variograms that increase and reach an asymptote indicate the existence of patches. The distance at which this asymptote is reached represents the distance at which the autocorrelation ceases and the average diameter of the patch in which the variable is aggregated. To assess if there was a significant patchy structure and to estimate the average patch diameter, we fitted the experimental variograms with theoretical variograms using weighted least squares^16,17^. This strategy is recommended for irregular spatial distributions of sampling points due to the variability that results in the number of pairs available per lag^16,45^. The theoretical models fitted to the variograms were:

6 $${\rm{Spherical}}:y(h)={C}_{0}+{C}_{1}[1.5\frac{{\rm{h}}}{{\rm{a}}}-0.5{(h/a)}^{3}]$$

if h ≤ a; y(h) = C if h > a

7 $${\rm{Gaussian}}:{\rm{y}}(h)={C}_{0}+{C}_{1}[1.5\frac{{\rm{h}}}{{\rm{a}}}-0.5{({h}^{2}/{{\rm{a}}}^{2})}^{3}]$$

8 $${\rm{Exponential}}:y(h)={C}_{0}+{C}_{1}[1-exp(\,-\,3h/a)]$$

9 $${\rm{Nugget}}\,({\rm{null}}\,{\rm{model}}):y(h)={C}_{0}$$

In the exponential and spherical models, the *sill* is the semi-variance reached when the variogram reaches the asymptote denoted by (*C*_0_ + *C*_1_). *C*_0_ represents the nugget effect or ordinate at the origin and *C*_1_ is the increment in semi-variance from *C*_0_ up to the point at which the variogram reaches the asymptote. For spherical models *a* is the interval, and for the exponential models the interval is taken when the semi-variance reaches 95% of the asymptote^17^. We only considered those models that explained at least 30% of the variance (R^2^ ≥ 0.3) as plausible and chose the theoretical model with greater explanatory power (R^2^). We took this proportion of explained variance as a proxy of the degree of space structuring for each variable^16^.

### Linear modeling of food availability and food set selection

We analyzed the explanatory power of mature fruits and young leaves availability on food set selection (per group) using linear models. We only modeled those seasons when food set was structured in space. Position in space (*x* + *y*) of each observation was included in the initial models as explanatory variables to test for the presence of gradients not explained by either availability index. We simplified these models comparing the ΔAIC of models including both explanatory variables together and separately, and chose the model with the lowest ΔAIC, which had a reduction of at least five units with respect to the null model^44^. During this simplification, we built experimental variograms with the residuals of each linear model and fitted theoretical models in the same way we did when we analyzed for *spatial structures*. We did this to determine (1) if they fulfilled the assumptions of spatial independence of linear models by not presenting spatial autocorrelation (nugget model as best fit) and (2) if the *spatial structures* (gradients and patches) found in SF disappeared in the residuals when using either of the availability indexes as explanatory variables. If the residuals did not show spatial patterns when the model included the variable, but they did when it excluded it, we considered that the variable explained both the mean variation (and slope) and the spatial variation of SF. If the residuals had spatial structures, we considered that the variable only explained the mean variation of SF^16^. If we still detected a spatial signal in the residuals of the adequate minimum linear models, we proceeded to construct generalized least squares (GLS) models. GLS are linear models that directly include in the residuals a spatial covariance structure through a variance-covariance matrix that considers the *range* and *sill* of a variogram function fitted to the residuals^16^. When considering the spatial structuring of the residuals, GLSs allow an adequate estimation of the parameters associated to the explanatory variables, without overestimating degrees of freedom, while estimating the size of the patches not explained by the model.

We additionally constructed linear models of the selected food explained by food availability for each item individually, but the saturated models were not simplified. Only the residuals where examined to confirm that there was no spatial structure being masked by a strong influence of explanatory variables^16^. All the analyses were carried out in R, v. 2.15.3 (http://R-project.org/). We used the geoR package for variography^46^, and the nlme package for linear models and GLS^47^.

### Ethical approval

All applicable international, national, and/or institutional guidelines for the care and use of animals were followed. All procedures performed in studies involving animals were in accordance with the ethical standards of the institution or practice at which the studies were conducted. This research was approved by SEMARNAT (SGPA/DGVS/10426/14), the governmental institution that regulates animal research, and complied with the laws of the country of Mexico.

## Results

### General description of food availability and selection

**Figure 2:**
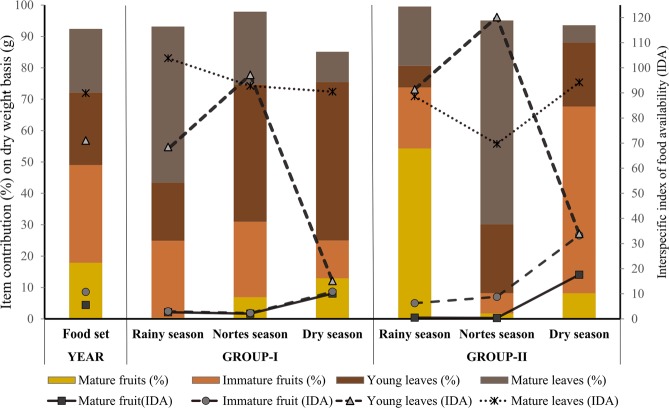
Item contribution to the diet and item availability per howler monkey groups/seasons. Bars represent the contribution **(**percentage on dry weight-basis) of fruits and leaves to the diet. Connecting lines and markers represent the mean value of interspecific index of food availability per items/season.

Both groups of howlers spent most of their time resting (77.3% G-I; 76.9% G-II), followed by feeding (137.9 hr: G-I = 16.3%, G-II = 15.8%), traveling (48 hr: G-I = 4.8%, G-II = 6.4%), and other behaviors (10.6 hr: G-I = 1.6%, G-II = 0.9%). Fruits (49.1%) and leaves (43.4%) constituted the bulk of their diet, but consumption varied between groups and seasons (Total mass: G-I = 6911.8 g; G-II = 9263.5 g) (Fig. 2; Supplementary Table S1). Leaves were more available than fruits throughout the year at both sites (Fig. 2; see median, min and max values of IFA and IDA in Supplementary Table S2). Mature leaves were constantly available, while young leaves and fruit exhibited seasonal variation, with fruit exhibited a peak in availability during the dry season.

### Spatial structuring of food availability and selection

**Figure 3:**
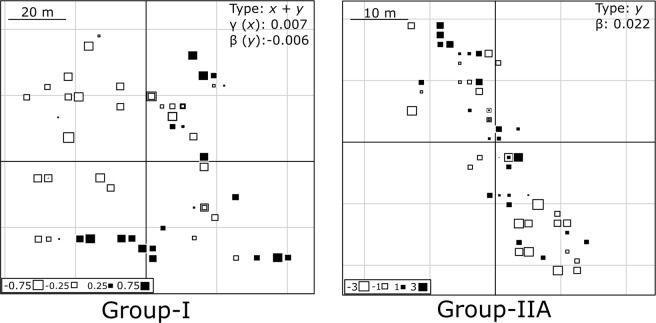
Spatial gradients represented by bubble maps of mature fruit availability (IFA|dry-season) in the study fragments. The squares are each tree, the symbol size is proportional to the observation’s deviation from the mean. Black and white squares indicate values above and below the mean, respectively. The type of gradient (*x* and *y*), direction (sign) and slope (γ and β).

Foods were aggregated in patches nested in gradients in seven combinations out of the 21 with proven spatial structuring, suggesting two scales of spatial structuring for a few availability combinations. Two combinations showed gradients without patches (only in site G-IIB), and 12 combinations had patches without gradients. The degree of spatial structuring, denoted by R^2^ of the theoretical model of the variogram, depended on the food item and season (parameters estimated in Table 1 and variograms in Supplementary Fig. S3, parameters of unstructured variables in Supplementary Table S4). In seven cases out of nine combinations with gradients, food items were along a north-south direction (*y)* and the remainder with east-west gradients (*x*). The intraspecific availability index (IFA) in G-I site showed east-west gradients *(x)* in mature leaves in the “nortes” season and young leaves in the rainy season, and *x* and *y* gradient in mature fruit in the dry season (see the bubble maps representing a gradient in Fig. 3). Only a north-south gradient was found for the interspecific availability index (IDA) in immature fruits in the dry season. For the G-II site, the IFA showed a north-south gradient for mature fruits in the dry season (Fig. 3), young leaves in “nortes” season, and immature fruits for the whole year. The IDA showed this same gradient in mature fruits in the dry season and for the whole year.

The variographic analysis illustrated that food availability was frequently aggregated in patches (19 combinations, 16 for fruits). Neither mature nor immature leaves showed a consistent aggregation pattern between sites, and leaves were aggregated in patches only at site G-I in two seasons. Availability indices of young leaves were not aggregated during the season of higher availability, “nortes” season, and were only aggregated in the rainy season (mean patch diameter of 15 m, R^2^ = 0.5). According to IFA, availability of mature leaves was only structured in the G-I site in patches of 11.9 m in the “nortes” season, and according to IDA in patches of 17.5 m in the rainy season, with a similar aggregation intensity (R^2^ = 0.3 and 0.4, respectively). Mature fruit patches occurred only in the dry season for both fragments and three sites (Table 1). The dry season had the highest availability of mature fruit (Fig. 2), we found high degree of aggregation according to both indices (IFA: R^2^ = 0.3–0.7, IDA: R^2^ = 0.3–0.5). According to IFA, the mean mature fruit patch diameter was 18 m for the G-I site, 19.5 m for the G-IIA site, and 40.9 m for the G-IIB site. In contrast, IDA showed a mean patch diameter of 26.1 m for the G-I site, 18.9 m for the G-IIA site, and 37 m for the G-IIB site. Immature fruits were aggregated according to IFA in the G-I site during the dry season in patches of 57 m and of 6.7 m according to IDA. Furthermore, when we analyzed immature fruit availability throughout the year, they were aggregated according to IFA in patches of 20 m only in G-IIA, and according to IDA in patches of 17 m in G-IIA and 41 m in G-IIB. In summary, food was structured in patches more than in gradients and mature fruits in the dry season were very patchily distributed. For mature fruits, we found similar mean patch size values according to both availability indices.

We detected that during the dry season food set selection was aggregated with a mean patch diameter of 14 m (R^2^ = 0.3) in the site G-I and of 6.9 m (R^2^ = 0.3) in the site G-IIA. The mature leaves were only aggregated in patches in the G-I site, where patches of mean 10.3 m were found in the rainy season (R^2^ = 0.3) and of mean 74.1 m in the dry seasons (R^2^ = 0.4). The mature leaf patches occurred within a North-South gradient. However, the majority of individual food item combinations (site and season) were not structured in patches or gradients (Table 1).

### Linear modeling: Food set selection explained by mature fruits availability

**Figure 4:**
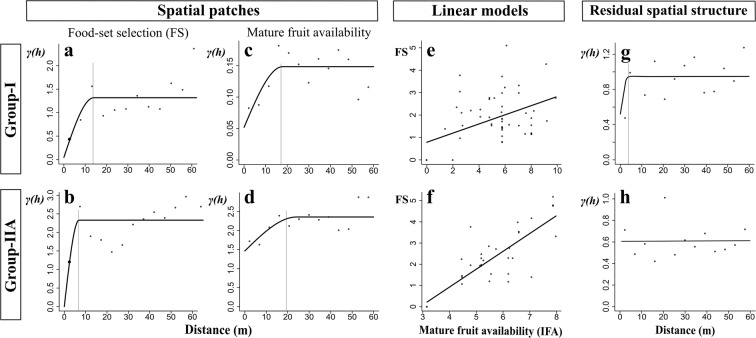
Spatial patches and linear models of food set selection that were explained by mature fruit availability of howler monkey groups. Variograms of food set selection (FS: **a**,**b**) and intraspecific index of food availability (IFA) of mature fruits (**c**,**d**) in dry season of black howler monkey groups. Generalized lineal squared model for G-I site (**e**) and lineal model for G-IIA site (**f**) (parameters in Table 2). (**g**) and (**h**) are the variograms of model residuals. Only the G-I site presented spatial structured residuals (variogram model: spherical; range = 3 m; nugget = 0.55), for G-IIA site residuals were not structure (variogram model: nugget). G-IIB site does not figure because there was not spatial structure in food selection variables. Gray line indicates the distance at which the autocorrelation ceases and the average diameter (in meters) of the patch. Fitted models are in solid black lines. *γ(h)* = semivariance axis. IFA-intraspecific index of food availability = $\overline{PC}\times {\rm{DBH}}$, where $\overline{PC}$ is the average of the phenological scores. FS: Food set selection (leaf and fruit intake on dry-weight basis).

Food set selection in G-I and G-IIA was aggregated and explained by mature fruit aggregation, but only according IFA. As described above, using variograms we first detected that the food set selection in the dry season was aggregated in patches in G-I and G-IIA sites (mean diameter of 14 and 6.9 m; Fig. 4a,b, respectively), but not in G-IIB, as well as mature fruit availability (mean diameter of 18 and 19.5 m, Fig. 4c,d). Subsequently, in the G-I site, linear modeling showed that once the mean variation of the food set variable was explained by mature fruit availability (Fig. 4e; GLS model parameters in Table 2), smaller patches persisted in the residuals (3 m mean diameter, Fig. 4g). For the G-IIA site, mature fruit availability also explained the patch aggregation associated to food set (Fig. 4f, parameters of the LM model in Table 2); however, the residuals of the model did not show any additional spatial structure (nugget model: Fig. 4h).

**Table 2:** Summary of linear models and generalized least squares model (GLS) of food set selection explained by mature fruits and young leaves.

Full model [Food set] ― [Food availability]		IFA	IDA	AIC Selected model	ΔAIC
	α (int)	β	γ		
**Group-I**					
4√[Food set_Dry] ― [Mature fruits_Dry] *	1.26	0.01	ni	168.2	7
4√[Food set_Dry] ― [Young leaves_Dry]	ni	ni	ni	178.9	0
Log10[Food set_Year] ― [Mature fruits_Year]	ni	ni	ni	179.5	0
Log10[Food set_Year] ― [Young leaves_Year]	ni	ni	ni	180.5	0
**Group-IIA**					
4√[Food set_Dry] ― [Mature fruits_Dry] *	−2.26	0.81	ni	96	43
4√[Food set_Dry] ― [Young leaves_Dry]	ni	ni	ni	195	0
4√[Food set_Nortes] ― [Mature fruits_Year]	ni	ni	ni	174.5	0
4√[Food set_Nortes] ― [Young leaves_Year]	ni	ni	ni	169.5	0

IFA-intraspecific index of food availability = $\overline{PC}$ × DBH, where $\overline{PC}$ is the average of the phenological scores. IDA: Interspecific index of food availability = $\overline{PC}$ × IVI, where IVI is the importance value index of trees. Food set in grams of dry weight (leaf and fruit intake, i.e. the sum of all items consumed). α, β and γ denote the estimate parameters for intercept, IFA and IDA indexes, respectively. ΔAIC = AIC_null_ - the AIC_selected model_ (AIC_null_ is the AIC of the response variable explained by its mean). ni = not included in the model after model selection. *GLS: Generalized least square model; a linear model with a spatial structure fit to the residuals; Saturated model includes IFA, IDA, *x* and *y* (north-south and east-west coordinates, respectively); Spatial and model graphs are in the Fig. 4.

## Discussion

We confirmed Hypothesis 1 by documenting that mature fruit productivity was aggregated in patches and this occurs in the peak season of availability. Furthermore, as suggested by the presence of gradients, mature fruits are also structured in a spatial pattern broader than the fragment size. The mean patch diameter associated to food set, leaf and fruit intake combined, during the dry season shows a diameter (6.9–14 m), which is in the same range of that associated to the mature fruits (18–19 m). This confirms Hypothesis 2. As predicted in Hypothesis 3, the spatial structure and mean variation of food set selection was explained by mature fruit patch structure, and not by their gradients. These results show that food selection by this primate is driven by the spatial distribution of fruits during certain seasons at small scale.

Trees showed intra and inter-specific variation in item productivity among seasons. The spatial modeling of both availability indices indicated that mature fruit was structured in space in the fragments during the same season. Hence, fruit productivity was aggregated, both due to the effect of large trees and to the influence of dominant plant species in the fragments. Supporting our first hypothesis, fruit production was structured in patches in the months of greatest production. However, when the availability was analyzed collapsing the seasonal values on a year basis, the intensity of aggregation was higher. This suggests that the aggregation of fruit availability sustains a certain degree of location stability throughout the year but fluctuates in intensity of production. It is also important to analyze both tree size and the contribution of dominant species in the analyses of resource spatial structures. The most dominant species were not equally dominant suppliers of fruits, except for *Sabal mexicana* (Supplementary Table S1). The most important species according IVI index in the G-I site were *Cupressus lindleyi* and *Albizia leucocalyx*; however, they did not represent a large part of the monkey’s diet (3.1%.and11.9%, respectively). Likewise, the most important species in the G-II site, were *Haematoxylon campechianum* and *Sabal mexicana*, but only the second one was a strongly used as food resource. It is possible that the fruit patch structuring is due more to an aggregated distribution of the individual trees bearing fruits and less to the fruiting synchrony among dominant species or even among individuals of the same species. This is supported by the frequent IFA spatial aggregation and subtler and less frequent aggregation of IDA. Moreover, some species have an asynchronous production and are uncommon, such as *Maclura tinctoria* at G-I site, but they can be a key resource for these monkey groups in periods of scarcity^41^. Likewise, the patchy spatial pattern associated to fruit availability may be a response to habitat loss^1^. In our study site, the conversion of forests to pastures and crops in the last 50 years reduced native vegetation by 90%. This modified the spatial distribution of large trees, and thus the availability of food resources for howler monkeys^48^.

It has been suggested that the availability, distribution, and quality of the feeding patches have an important effect on primate social organization, group cohesion, and food choice^34,49,50^. Many primate species exhibit spatial knowledge of the environment and seemingly plan and travel routes to find a given resource area^51^. The concept of food patches is therefore central to most models of primate socioecology^52^. The definition of a food patch has always been controversial. This is largely because it has been done using different criteria and based on the observations of animal behavior^31,32^. Several studies have supported the use of DBH as an indicator of plant productivity and demonstrated that it is a good estimate of fruit abundance, hence it is used as an estimate of food patch size^20^. Alternatively, patches are defined as a continuum of food arranged in such a way that the forager can feed without interruption and may occur in an isolated tree or in a group of trees with contiguous canopies^31,53,54^. These are convenient operational definitions; however, these definitions result in a patch being defined according to the specific primate being studied and the specific questions addressed^8,55^. This is especially true when primates tend to spread out and simultaneously feed from multiple trees^55^. Consequently, other studies have attempted to improve upon traditional patch definitions by incorporating the individual perspective^8^, such as the focal-tree method. Some of them include spatial analyses to define food patches using grid cells^55^. Expanding the way in which a feeding patch is evaluated using the animal’s perspective and linking this information to the spatial arrange of the resource, results in a more accurate assessment of the relationship between foragers and food distribution.

Under the traditional definition of food patch (DBH), it has been observed in black howlers that time spent feeding in a patch did not correlate with patch size^35^. In contrast, in mantled howler monkeys *(A. palliata*), there is a strong relationship between feeding time and the size of the fruit foraging patch^31,56^. However, this evidence does not allow to address whether the limits associated to foraging patches and its aggregation coincide with those of food items because these concepts have not been spatially and explicitly operationalized before^32^. The present study estimates patch diameters independently, one for the food resource and other for the food selection, based on modeling spatial autocorrelation. In support of our second hypothesis, the average patch diameter associated to local fruit availability (IFA 18–19 m) may represent several tree crowns and was assessed independently of the patch size associated to the food selection (6.9–14 m). This finding supports previous evidence that food selected by howlers often occur in clusters or patches and that they exploit larger fruit patches possibly due to its grouping behavior^53^. Similar conclusions have been described for other primates, such as black spider monkeys (*Ateles paniscus chamek*) and muriquis (*Brachyteles amchnoides*)^57,58^. Our study shows how an independent spatial modeling strategy of animal resources and behavior is able to operationalize the definitions of food patches and foraging, estimating the scales in which both are structured, and allowing their independent variation. This approach also subsequently allows testing hypotheses on the explanatory power of resource spatial distribution on behavior.

The spatial structuring and slope associated to food set selection by howler monkeys is explained by the availability of one of the most preferred and limiting foods for primates, mature fruits (Hypothesis 3). This pattern occurred for both groups when fruit production was high, but not for young leaves. The howler’s diet is not typically rich in lipids, but some fruits have high lipid content, which likely plays an important role in their food choice^59^. Although the spatial relationship has not been addressed in this way, previous studies have also observed that the consumption of fruits and flowers shows a positive correlation with the availability of fruits for howler monkeys^28,60^ and in other primates such as gibbons (*Hylobates lar*)^61^ and chimpanzees (*Pan troglodytes*)^62^.

Changes in the timing and intensity of fruit production have effects on the structure and function of the ecosystem^19^. For example, the density of frugivorous animals is negatively affected by a strong seasonality in the availability of fruits^18^. However, large animals (>5 kg) may be more tolerant to highly seasonal resources than smaller ones^63^. Studying the spatial structuring of food resources and evaluating whether the forager follows the preferred or limiting food could help explain this tolerance. Our findings suggest that for black howlers the tolerance to resource seasonality is due in part to the fact that they exploit the preferred or limiting food when it is available. However, the mixed consumption of leaves and fruits^59^, along with their small gut capacity^64^, prevent these primates from consuming large quantities of a food in a single day. Instead, they concentrated in consuming other foods in small quantities in the area where the preferred food is aggregated, probably according to its nutrient content, hence the spatial association in a food set basis. Several primates have a mixed foraging strategy in which some foods are encountered when animals travel. However, most of this movement is driven by the need to exploit a known set of patches in a near-optimal order^51^. It is noticeable that the food set selection exhibits spatial structuring, but when each item consumed is analyzed separately there is no spatial structuring pattern. The spatial statistics methods used here are very data demanding^16^ and possibly the effect size when separating fruits and leaf data was not enough to detect the expected itemized spatial structures.

It has been suggested that the intraspecific variation in the nutritional content of the available food can generate spatial aggregation on a very small scale (i.e., between trees). This could further influence the spatial structuring of food selection by primates within available patches^26^. Such micro-structuring could be illustrated in our study by the residuals of the GLS model of the analysis of spatial dependence between food set selection and fruit availability were structured in smaller patches (3 m) in the G-I site. The residual spatial autocorrelation of a GLS model suggests the potential relevance of an explanatory process structured in space that was not previously considered^65^. This facilitates the generation of new ecological hypotheses. For example, the fact that the patch diameter associated to the food set was smaller than those of mature fruits, suggest that the monkeys select the trees, or crown zones, with the desired nutritional content within the patches where that resource is available.

It is essential to find the relevant scale to spatially study the relationships between animals and their environment because different processes are often at work, depending on the scale^66^. In some primates, biological processes are associated with specific scales^67,68^. For example, the population density of howlers studied at two spatial scales, 100 and 500 ha, is better explained by fragment size on a smaller spatial scale than on larger ones^9^. Consequently, conclusions derived from a spatial scale often cannot be extrapolated to others^66^. Our results suggest that feeding behavior of howlers operates at a small scale within the food patches and not at broader scales since the food selection did not follow the spatial pattern in gradients presented by the resources. It is known that fruiting changes are more pronounced at small scales due to seasonal and interannual variability, or to abnormally high fruiting of individual plants^21^. In addition, there is evidence that seasonal variation in the spatial availability of food supports environmentally driven changes in primate movement patterns^69^.

It is worth noting that neither food availability, nor selection of young and mature leaves, during the seasons of higher production, were structured in space. However, a spatial aggregation associated to the mature leaf availability in the rainy season was found. In general, leaves are more abundant and more evenly distributed than fruits, therefore, folivorous primates generally spend less time searching for food, rest more, and occupy smaller areas than frugivorous and insectivorous species^11,70^. Although there are reports in other howler monkey species (e.g., *A. seniculus*) that food availability and the consumption of leaf parts are correlated^71^, our results show that the howlers preferentially feed on the trees that have marked fluctuations in resource availability. That may be the reason for their differing patterns of leaf’ ingestion between localities, seasons, and years^35,64^. For example, for mantled howler monkeys (*A. palliata*) in coffee plantations in Nicaragua, the seasonal pattern of food selection did not follow the production pattern of the more abundant phenophases (i.e., mature leaves), nor of the most limiting resources (i.e. fruits)^56^. Hence, foods are consumed selectively, even though there are abundant potential resources in the habitat which are ignored.

In conclusion we found that there is a spatial aggregation of food resources at two scales, in patches nested in gradients, mainly fruits, and that this spatial structuring depends on the season. The spatial distribution in availability gradients had no explanatory power over the spatial distribution of food selection by howlers. However, the variance of food set selection was explained by the availability of mature fruit and the spatial structure of the food set selection was explained by the patchy distribution of fruit within the fragments during the season of higher production. Thus, howler monkeys probably base their foraging decisions on local spatial scales influenced by clusters of fruiting trees. This may maximize leaf and fruit intake in response to spatial and seasonal aggregation of the preferred resource.

## Supplementary information

Supplementary Information

Dataset 1

## Data Availability

The datasets generated during and/or analyzed during the current study are available into the manuscript and the supplementary material.
