# Supplementary material for: Spatial aggregation of fruits explains food selection in a neotropical primate (Alouatta pigra)
Source: Sci Rep. 2019 Dec 19;9:19452. doi: 10.1038/s41598-019-55932-y (PMC6923416; doi:10.1038/s41598-019-55932-y)
Supplement: Supplementary file 1 — Supplementary Information [file 41598_2019_55932_MOESM1_ESM.pdf]

Supplementary information

Title: Spatial aggregation of fruits explains food selection in a neotropical primate (*Alouatta pigra*)

Authors: John F. Aristizabal, Simoneta Negrete-Yankelevich, Rogelio Macías-Ordóñez, Colin A. Chapman, Juan C. Serio-Silva

Table S1. Tree species representing at least 1% of *Alouatta pigra* diet in terms of time invested and dry-weight ingested, and the importance value indices (IVI) of tree species.

Tree specie	Group-I			Group-II			% Min Total	% DW Total
	% Minutes	% Dry-weight	IVI	% Minutes	% Dry-weight	IVI		
Albizia leucocalyx	18.2	11.9	41.7	33.2	12.0	1.9	25.6	11.9
Coccoloba barbadensis	0.0	0.0	---	0.6	1.0	1.0	0.3	0.5
Ceiba pentandra	4.7	4.8	2.5	0.0	0.0	1.0	2.4	2.0
Chrysophyllum cainito	0.0	0.0	---	0.2	1.3	0.5	0.1	0.8
Cupressus lindleyi	3.4	3.1	64.6	0.0	0.0	---	1.7	1.3
Guazuma ulmifolia	4.0	7.2	3.8	1.0	1.9	15.4	2.5	4.2
Hematoxylon campechianum	0.8	0.4	2.5	14.8	3.2	48.6	7.8	2.0
Inga edulis	0.0	0.0	---	1.8	4.4	1.0	0.9	2.5
Lonchocarpus castilloi	4.2	2.3	2.5	1.0	0.7	1.9	2.6	1.4
Maclura tinctoria	22.4	15.6	3.8	10.2	5.3	1.9	16.4	9.7
Mangifera indica	0.0	0.0	---	7.2	23.4	2.9	3.6	13.4
Manilkara zapota	0.0	0.0	---	2.0	0.9	1.0	1.0	0.5
Parmentiera aculeata	0.0	0.0	---	5.9	7.5	0.5	2.9	4.3
Pithecellobium lanceolatum	28.3	32.1	11.4	4.3	4.0	1.9	16.4	16.0
Sabal mexicana	1.6	4.1	11.4	6.4	24.1	42.5	4.0	15.6
Spondias purpurea	0.0	0.0	---	1.2	3.3	0.5	0.6	1.9
Tabebuia rosea	0.8	2.3	3.8	1.6	1.4	4.3	1.2	1.8
Zanthoxylum riedelianum	3.3	2.6	16.3	0.0	0.0	---	1.7	1.1

---: not present in the fragment. Lianas contribution to the diet: 1) *Melothria pendula*: Group-I: time= 0.8% and dry-weight= 1.6%; Group-II: time= 3.4% and dry-weight= 2.8%. 2) *Syngonium podophyllum*: Group-I: time= 7.3% and dry-weight= 11.2%; Group-II: time= 1.1% and dry-weight= 1.1%.

Table S2. Food availability given by Intraspecific index of food availability (IFA) and Interspecific index of food availability (IDA).

Group/Item	Intraspecific index			Interspecific index		
	Median	Min	Max	Median	Min	Max
**GROUP-I**						
Mature leaves	72.7	18.2	531.8	128.0	3.1	194.4
Young leaves	46.6	14.0	229.1	61.8	1.3	140.9
Mature fruits	7.3	0.8	76.4	5.9	0.1	42.1
Immature fruits	6.9	0.9	98.2	8.8	0.5	36.1
**GROUP-II**						
Mature leaves	84.4	0.1	487.6	78.0	0.1	168.9
Young leaves	90.0	0.1	332.2	91.1	0.8	131.3
Mature fruits	3.7	0.1	65.5	3.1	0.1	34.8
Immature fruits	9.3	0.1	82.7	5.3	0.1	65.7

Figure S3. Variograms of Selected food (SF), IFA-intraspecific index of food availability and IDA: Interspecific index of food availability that presented a spatial structure in two groups of black howler monkeys. A and B correspond to the Group-II fragment division (North and south, respectively).

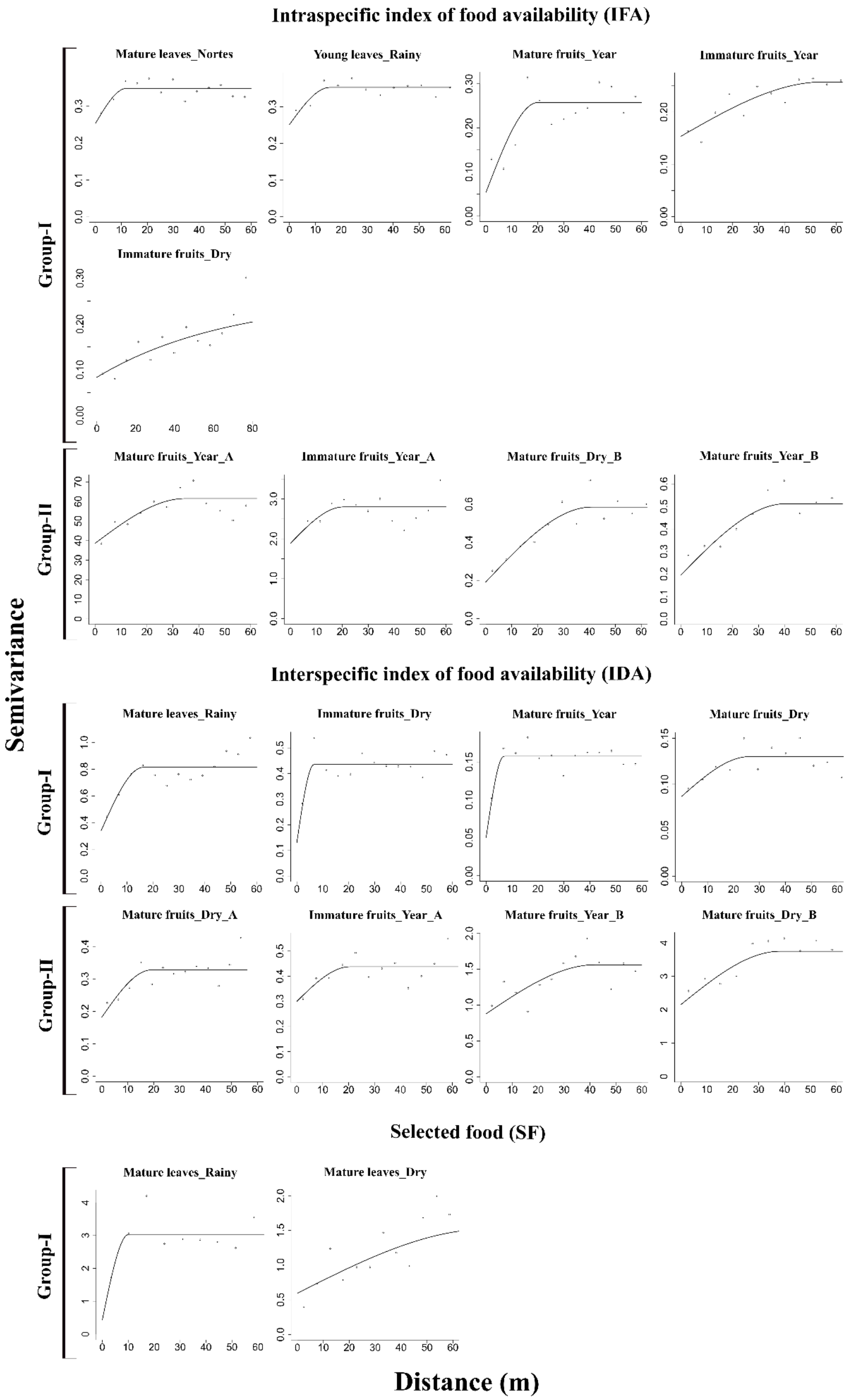

Table S4. Parameters of not significative variograms of the studied variables of black howler monkey groups.

Group/Item Season	Transformation	Gradient	Variogram model				
			Model	Range	Nug	Sill	R2
**GROUP-I**							
**IFA-Mature leaves**							
Rainy	√Y+c	ns	Spherical	21.5	0.004	0.008	0.2
Dry	Log10	X	ns	ns	ns	ns	ns
**IFA-Young leaves**							
Nortes	√Y+c	ns	Spherical	10.7	0.003	0.007	0.2
Dry	3√Y	ns	ns	ns	ns	ns	ns
**IFA_Immature fruits**							
Nortes	Log10	ns	ns	ns	ns	ns	ns
**IDA-Mature leaves**							
Rainy	3√Y	ns	Spherical	23.3	0.3	0.8	0.2
Nortes	3√Y	ns	ns	ns	ns	ns	ns
**IDA-Young leaves**							
Nortes	3√Y	ns	ns	ns	ns	ns	ns
Dry	3√Y	ns	ns	ns	ns	ns	ns
**IDA_Immature fruits**							
Year	√Y+0.0005	ns	ns	ns	ns	ns	ns
Nortes	3√Y	ns	ns	ns	ns	ns	ns
**SF_Food set**							
Rainy	log10	ns	ns	ns	ns	ns	ns
**SF_Mature fruits**							
Nortes	log10	ns	ns	ns	ns	ns	ns
**SF_Young leaves**							
**GROUP-II**							
**IFA_Mature leaves_A**							
Rainy	Log10 + 0.1	ns	ns	ns	ns	ns	ns
**IFA_Mature leaves_B**							
Rainy	4√Y	ns	ns	ns	ns	ns	ns
Nortes	Log10 + 0.1	ns	Spherical	11.9	0.36	0.47	0.2

**IFA_Young leaves_A**							
Rainy	Normal	ns	ns	ns	ns	ns	ns
Dry	2√Y+c	ns	ns	ns	ns	ns	ns
**IFA_Young leaves_B**							
Rainy	3√Y	ns	ns	ns	ns	ns	ns
**IFA_Immature fruits_B**							
Dry	3√Y	ns	ns	ns	ns	ns	ns
**IDA_Young leaves_A**							
Rainy	3√Y	ns	ns	ns	ns	ns	ns
**IDA_Young leaves_B**							
Rainy	3√Y	ns	ns	ns	ns	ns	ns
Dry	3√Y	ns	ns	ns	ns	ns	ns
**IDA_Mature leaves_B**							
Rainy	3√Y	ns	ns	ns	ns	ns	ns
Nortes	3√Y	ns	ns	ns	ns	ns	ns
Dry	3√Y	X	Spherical	15.4	0.98	1.16	0.2
**IDA_Mature fruits_A**							
Year	Log10 + 100	ns	ns	ns	ns	ns	ns
**IDA_Immature fruits_B**							
Year	3√Y	ns	ns	ns	ns	ns	ns
Dry	3√Y	Y	Exponential	55	0.59	0.87	0.2
**SF_Food set_A-B**							
Year	Log10 + 0.1	ns	ns	ns	ns	ns	ns
Rainy	Log10 + 0.1	ns	ns	ns	ns	ns	ns
**SF_Mature leaves_A-B**							
Year	Log10 + 0.1	ns	ns	ns	ns	ns	ns
Nortes	3√Y	ns	ns	ns	ns	ns	ns
**SF_Young leaves_A**							
Year	Log10 + 0.01	ns	ns	ns	ns	ns	ns

IFA-intraspecific index of food availability = $\overline{PC}$*DBH, where $\overline{PC}$ is the average of the phenological scores. IDA: Interspecific index of food availability= $\overline{PC}$*IVI, where IVI is the importance value index of trees. SF= Selected food (grams of dry-weight). A and B correspond to the Group-II´s fragment division (North and south, respectively).
